# Chemical composition, vasorelaxant, antioxidant and antiplatelet effects of essential oil of *Artemisia campestris* L. from Oriental Morocco

**DOI:** 10.1186/s12906-017-1598-2

**Published:** 2017-01-31

**Authors:** Ikram Dib, Marie-Laure Fauconnier, Marianne Sindic, Fatima Belmekki, Asmae Assaidi, Mohamed Berrabah, Hassane Mekhfi, Mohammed Aziz, Abdelkhaleq Legssyer, Mohamed Bnouham, Abderrahim Ziyyat

**Affiliations:** 1 Laboratoire de Physiologie, Génétique et Ethnopharmacologie URAC-40, Département de Biologie, Faculté des Sciences, Université Mohammed Premier, Oujda, Morocco; 2 Unité de Chimie Générale et Organique, Gembloux Agro-bio Tech, Université de Liège, Gembloux, Belgium; 3 Laboratoire Qualité et Sécurité des Produits Alimentaires, Gembloux Agro-Bio Tech, Université de Liège, Gembloux, Belgium; 4 Laboratoire de Chimie du Solide Minéral et Analytique, Département de Chimie, Faculté des Sciences, Université Mohammed Premier, Oujda, Morocco

**Keywords:** *Artemisia campestris* L, GC/MS, Antioxidant, Antiplatelet, Vasorelaxant

## Abstract

**Background:**

*Artemisia campestris* L. (Asteraceae) is a medicinal herb traditionally used to treat hypertension and many other diseases. Hence, this study is aimed to analyze the essential oil of *A. campestris* L (AcEO) and to investigate the antiplatelet, antioxidant effects and the mechanisms of its vasorelaxant effect.

**Methods:**

The chemical composition of AcEO was elucidated using GC/MS analysis. Then, the antioxidant effect was tested on DPPH radical scavenging and on the prevention of β-carotene bleaching. The antiplatelet effect was performed on the presence of the platelet agonists: thrombin and ADP. The mechanism of action of the vasorelaxant effect was studied by using the cellular blockers specified to explore the involvement of NO/GC pathway and in the presence of calcium channels blockers and potassium channels blockers.

**Results:**

AcEO is predominated by the volatiles: spathulenol, ß-eudesmol and p-cymene. The maximal antioxidant effect was obtained with the dose 2 mg/ml of AcEO. The dose 1 mg/ml of AcEO showed a maximum antiplatelet effect of, respectively 49.73% ±9.54 and 48.20% ±8.49 on thrombin and ADP. The vasorelaxation seems not to be mediated via NOS/GC pathway neither via the potassium channels. However, pretreatment with calcium channels blockers attenuated this effect, suggesting that the vasorelaxation is mediated via inhibition of L-type Ca^2+^ channels and the activation of SERCA pumps of reticulum plasma.

**Conclusion:**

This study confirms the antioxidant, antiplatelet and vasorelaxant effects of *A.campestris* L essential oil. However, the antihypertensive use of this oil should be further confirmed by the chemical fractionation and subsequent bio-guided assays.

## Background

*A. campestris* L. is an Asteraceae plant commonly known as field wormwood; it is a perennial undershrub (30–150 cm height), with branched and ascending brownish-red stems. Leaves are green, the basal are 2–3 pinnatisects, the upper are simple. Inflorescence is an ovoid, heterogamous yellowish capitulum, with an involucral bracts; ray flowers are female, pistillate and fertile, while the disk flowers are sterile, and functionally male [1–3]. In Morocco, *A.campestris* L. known as “Allal”, is used as antidiabetic [4], to treat digestive, respiratory, metabolic, allergic problems [5, 6] and cutaneous problems [7]. This herb has many other ethnomedicinal uses like antihypertensive [8], emmenaguogue [9, 10], and well known for treatment of liver and kidney disorders [11–13] and as. Previous pharmacological studies proved that *A. campestris* L. possesses antioxidant [14–20], antibacterial, antifungal [20–25], insecticidal [26–28], antitumor [14, 29–31], antivenin [32, 33], hepatoprotective, nephroprotective [34–37] and antidiabetic [38, 39] effects. Recently a clinical trial conducted on volunteers demonstrated that *A.campestris* L. enhanced 33.3% to 50% decrease in arterial pressure among hypertensive smoker patients [40]. In another study, the administration of *A.campestris* L. extract induced antihypertensive effect on envenomed hypertensive rats, and provoked 10% to 30% of hypertension drop, while the pretreatment with the herb extract prevented the rise of hypertension [32]. Nevertheless, no in-vitro study has been elaborated to evaluate the antihypertensive potential of this plant. In the aim to highlight the importance of this plant in the cardiovascular therapy, this study was performed to analyze the essential oil of *A. campestris* L. (AcEO) growing in oriental Morocco, to investigate its vasorelaxant and subsequent mechanism of action and to determine its antiplatelet and antioxidant effects.

## Methods

### Chemicals

The following drugs and solvents were used in this study: (±)-verapamil hydrochloride (Sigma Aldrich, China), (R)-(-)-phenylephrine hydrochloride [Phe] (Sigma Aldrich, Germany), 1H-[1, 2, 4] Oxadiazolo[4,3-a]quinoxalin-1-one [ODQ] (Cayman Chemical, USA), 2, 2-diphenyl-1-picrylhydrazyl [DPPH] (Alfa Aesar, Germany), 4-aminopyridine [4-AP] (Alfa Aesar, Germany), adenosine 5′-diphosphate [ADP] (Sigma Aldrich, Germany), atropine (Sigma Aldrich, China), barium chloride dehydrate [BaCl_2_] (AnalaR Normapur - VWR International, Belgium), calcium chloride dehydrate [CaCl_2_, 2H_2_O] (Scharlau chemie, Spain), calmidazolium chloride (Sigma Aldrich, USA), carbamylcholine chloride [carbachol] (Sigma Aldrich, USA), citric acid (Farco chemical, Puerto Rico), D(+)-glucose anhydrous (Sigma Aldrich_Riedel-de Haen, Germany), gelatin extrapur (HIMEDIA, India), glybenclamide (Sigma Aldrich, USA), hydroxocobalamin hydrochloride (Fluka, USA), indomethacin (Sigma Aldrich-Fluka, Italy), L-ascorbic acid (Sigma Aldrich, UK), linoleic acid (Sigma Aldrich, USA), magnesium sulfate [MgSO_4_] (Sigma Aldrich, Germany), Nω-Nitro-L-arginine methyl ester hydrochloride [L-NAME] (Sigma Aldrich, Switzerland), potassium di-hydrogen phosphate [KH_2_PO_4_] (Panreac, Spain), Rp-8-Bromo-β-phenyl-1,N2-ethenoguanosine3′,5′-cyclicmonophosphorothioate sodium salt [Rp-8-Br-PET-cGMP] (Sigma Aldrich, Germany), sodium chloride [NaCl] (Sigma Aldrich_Riedel-de Haen, Denmark), sodium hydrogen carbonate [NaHCO_3_] (Farco chemical, Puerto Rico], potassium chloride [KCl] (Sigma Aldrich_Riedel-de Haen, Germany), tetraethyl ammonium chloride hydrate [TEA] (Sigma Aldrich, USA), thapsigargin (Sigma Aldrich, Israel), thrombin, from bovine plasma (Sigma Aldrich, USA), trisodium citrate (Acros organics, belgium), Tween 40 (Sigma Aldrich, USA), β-carotene (Sigma Aldrich, USA). The solvents utilized were: chloroform (Sigma Aldrich_Riedel-de Haen, Germany), diethyl ether (Sigma Aldrich, Germany), dimethyl sulfoxide [DMSO] (Sigma Aldrich_Riedel-de Haen, Germany), methanol (Sigma Aldrich, Germany). All chemicals and solvents used were analytical grade. The stock solutions of ODQ, thapsigargin and Rp-8-Br-PET-cGMP were prepared in DMSO whereas indomethacin was prepared in 5% (w/v) sodium bicarbonate solution. All other drugs were dissolved in distilled water.

### Plant material

The aerial part of *A. campestris* L. was collected at flowering stage in September 2012 in desert region of Figuig (in South-East of Morocco in the border area with Algeria). The species was identified by a botanist Pr. Aatika Mihamou from biology department, and a voucher specimen was deposited in the herbarium of the Faculty of Sciences, University Mohamed First (Oujda, Morocco) under the number HUMPOM-151.

### Preparation of *A.campestris* L. essential oil (AcEO)

About 2 kg of the air-dried plant were used. For the extraction of the essential oil, the stems were thrown and the rest of the plant (leaves and flowers) was subjected to the hydrodistillation for 4 h by using *Clevenger* apparatus. The obtained oil was yellowish with characteristic odor; it was then separated from the distillate and stored in sealed glass vial at 4 °C until the moment of analysis. The essential oil was obtained in a yield of 0.4% (w/w).

### Gas Chromatography (GC) analysis

The GC analysis of AcEO was performed on an Agilent 5973 N GC-MS coupled to an Agilent 6890 gas chromatograph fitted with an injector at 250 °C (Splitless mode) and equipped with an HP-5MS capillary column coated with 5% phenyl-methyl siloxane (30 m length × 0.25 mm internal diameter × 0.25 μm of film thickness, Agilent 19091S-433). The column pressure was set to 51.6 × 103 Pa. The oven temperature was held at 40 °C for 2 min, and then programmed to 250 °C at a rate of 8 °C/min. Helium was used as the carrier gas at a flow rate of 1.1 ml/min. Diluted sample in diethyl ether was manually injected.

### Gas-Chromatography-Mass Spectrometry (GC-MS) analysis

The GC–MS analysis of AcEO was performed on an Agilent 5973 N GC-MS coupled to an Agilent 6890 gas chromatograph fitted with an injector at 250 °C (Splitless mode) and equipped with an HP-5MS capillary column coated with 5% phenyl-methylsiloxane (30 m length × 0.25 mm internal diameter × 0.25 μm of film thickness, Agilent 19091S-433). The column pressure was set to 51.6 × 10^3^ Pa. The oven temperature was held at 40 °C for 2 min, and then programmed to 250 °C at a rate of 8 °C/min. Helium was used as the carrier gas at a flow rate of 1.1 ml/min. The mass spectra (MS) were operated in electron impact mode (70 eV) with the electron multiplier set at 1823.5 V, and the MS data were acquired in scan mode. The peaks were quantified by calculating the percentage of the peak area of each component by comparison to the sum of the peaks of other compounds. The identification of the components was performed on the basis of chromatographic comparison of the recorded retention time with computed mass-spectrum data libraries (Pal600K and Wiley275).

### β-Carotene bleaching assay

The β-Carotene bleaching test of AcEO was determined based on the standard procedure [41]. A volume of 1 ml of chloroform solution of β-carotene (0.1 mg/ml) was added to a round flask containing 20 mg of acid linoleic and 200 mg of Tween 40. Chloroform was completely removed at 40 °C under vacuum, and 50 ml of distilled water was slowly added and vigorously shaken. Three doses of AcEO and the reference standard Ascorbic acid (0.1; 1 and 2 mg/ml) were dissolved in methanol. Aliquots (200 μl) of AcEO and ascorbic acid were added to 5 ml of β-carotene/linoleic acid emulsion. A control preparation was obtained by adding 200 μl of methanol to 5 ml of β-carotene/linoleic acid emulsion. Absorbance of the preparations was measured at 490 nm before and after 2 h of incubation in a water bath at 50 °C. All trials were performed in triplicate. Antioxidative activity (AA %) in percentages was calculated using the following formula:

$$ \mathrm{AA}\%=\left[\frac{\left(\mathrm{As}120-\mathrm{Ac}120\right)}{\left(\mathrm{Ac}0-\mathrm{Ac}120\right)}\right] \times 100 $$

Wher Ac_0_ is the absorbance of the control respectively measured before the incubation. As_120_ and Ac_120_ are the absorbance of the test and the control respectively measured after 2 h of incubation.

### DPPH radical scavenging assay

The DPPH free radical scavenging activity of the EOs was evaluated as described by Senthilkumar et al. [42], with slight modifications. A volume of 1 ml of 0.1 mM of methanolic solution of the free radical 2,2-diphenyl-1-picryl-hydrazyl (DPPH) was added to 1 ml of increased doses of AcEO ranging from 0.1 mg/ml to 2 mg/ml previously dissolved in methanol. A control sample using methanol instead of AcEO was prepared. Ascorbic acid dissolved in methanol (0.1–2 mg/ml) was used as standard. The preparations were incubated for 30 min in obscurity at room temperature, then after, absorbance was measured at 517 nm. Pure methanol was used as blank. Measurements were carried out in triplicate for each experiment.

Antioxidant activity was calculated using the equation:

$$ \%\ \mathrm{scavenging}=\left[\frac{\left(\mathrm{Ac}-\mathrm{As}\right)}{\mathrm{Ac}}\right]\mathrm{x}100 $$

Where Ac is the absorbance of the control and As is the absorbance of the sample.

### Experimental animals

Wistar rats and albino mice were provided from the local colonies of department of Biology (Faculty of Sciences, Oujda-Morocco); they were maintained in standard conditions, with a photoperiod of 12 h light and dark, and they were allowed to free access of water and food. All animals were cared for in compliance with the Guide for the Care and Use of Laboratory Animals, published by the US National Institutes of Health (NIH) [43].

### Acute toxicity

Acute toxicity of AcEO was performed on the basis of the protocols conducted in previous similar studies [44, 45]. 25 albino mice were divided into five groups of five animals each. After 18 h of fasting with free access to water, the doses of 0.5, 1, 1.5 and 2 mg/kg of AcEO were given orally to the mice, using a solution of gelatin 5% as vehicle. The control group was fed with 0.5% gelatin solution. The use of gelatin as an emulsifier is attributed to its non-toxic and non-irritability qualities as carrier molecule [46], besides its hydrophobic character that makes this macroprotein preferably used in pharmaceutical application and food industry as natural emulsifier and stabilizer [47, 48]. Also, gelatin is largely used as biodegradable macromolecule largely used for encapsulation of essential oils, mainly utilized in pharmaceutical and cosmetics to prevent eventual decomposition, evaporation or oxidation of the volatile oils [49]. Indeed, Sutaphanit and Chitprasert (2014) demonstrated that there was no significant interaction between gelatin and basil essential oil, along the encapsulation process [50]. General behavior and mortality were observed permanently during the four hours succeeding the dosing, and occasionally during the first 24 h. The animals were monitored daily for any additional signs of toxicity and weekly for changes in body weight. Dead animals were sacrificed just after their death and the survived animals were sacrificed at the end of the experimentation by overdose of anesthesia by ethylic ether, and the organs were examined macroscopically for any toxicological alterations. The stomach was longitudinally incised by the greater curvature and ulceration or perforations of gastric mucosa were observed. The liver and kidneys have been weighted after been cleaned from connective tissues.

### Platelet aggregation assay

Wistar rats weighing 250–380 g were slightly anesthetized with ether. Abdominal aorta was catheterized and blood was collected in anti-coagulated tubes with a mixture of acid citric (130 mM)-trisodium citrate (170 mM)-glucose 4% (9:1, v/v). Washed platelets were prepared according to the experimental design reported by Gadi et al. [51]. A first centrifugation of blood (230 x g/15 min) was made and permitted to obtain a platelet rich plasma (PRP), which was centrifuged a second time (400 x g/ 15 min) to obtain the platelet pellet. The platelets were then washed once with washing buffer (NaCl 137 mM, KCl 2.6 mM, NaHCO_3_, 12 mM, MgCl_2_ 0.9 mM, glucose 5.5 mM, gelatin 0.25%, pH 6.5), centrifuged for the last time (400 × *g*/ 15 min) and suspended in the final buffer with the following composition (mM): NaCl 137, KCl 2.6, CaCl_2_ 1.3, MgCl_2_ 0.9, glucose 5.5, Hepes 5, gelatin 0.25%, pH 7.4) in order to have a final platelet concentration of 5 × 10^5^ cells/mm^3^.

Platelet aggregation was performed using an aggregometer (Chrono-Log, Havertown, PA, USA). In a special tube containing 400 μl of washed platelets at 37 °C with continual stirring at 1000 rpm, the aggregation was stimulated with aggregating agents, thrombin (0.1 U/ml) and ADP (1 μM). For test studies, platelets were preincubated with different concentrations of AcEO (0.1, 0.5 and 1 mg/ml) for 1 min in the cuvette before the stimulation by the aggregating agents cited above. In all experiments, the platelet aggregation was then recorded during 5 min. AcEO was dissolved in 0.5% DMSO. Control experiments demonstrated that the concentrations of DMSO had no significant effect on the aggregatory effect.

### Determination of the mechanism underlying the vasorelaxant activity of AcEO

The vascular tone was measured by referring to previously described procedure [52]. Wistar rats weighting 200-300 g were anesthetized with sodium pentobarbital (0.1 ml/100 g body weight). The thoracic aorta was quickly and gently removed, cleaned of adherent connective tissue and cut into rings (3-4 mm in length). Rings were gently introduced between two stainless-steel hooks and placed in organ chamber (emka technologies, Paris) containing 11 ml of Krebs solution gassed with 95% O_2_ and 5% CO_2_ and maintained at 37 °C and pH 7.4. One hook was connected to an isometric force transducer (emka technologies, Paris) and a tension of 1 g was applied to the vessels then they were allowed to stabilize for 30 min. The composition of Krebs solution was as follows (mmol/L): NaCl 119, KCl 4.7, CaCl_2_ 2.6, MgSO_4_ 1.2, KH_2_PO_4_ 1.2, NaHCO_3_ 25, and Glucose 11. Endothelial integrity was monitored by the percentage of relaxation evoked by carbachol (10^−4^ M) after a steady contraction was reached with phenylephrine (10^−6^ M). Rings with carbachol-induced relaxation less than 50% were discarded. AcEO was dissolved in final concentration of 0.5% DMSO. Control experiments showed that DMSO at 0.5% had no significant effect on the vascular tone.

### Vasorelaxant effect of AcEO on denuded aorta, and on intact aorta preincubated with Atropine and Calmidazolium

In endothelium-intact aorta (*n* = 6), steady tension was evoked by Phen (1 μM), then, AcEO (10^−4^–10^−1^ mg/ml) was cumulatively added to the Krebs solution. The contribution of endothelium, muscarinic receptor and subsequent Ca^2+^-CaM complex formation in the vasorelaxation-induced effect was checked out by the application of experiments described by Monteiro et al. [46]. Denuded rings (*n* = 6) were obtained by gentile rubbing of the lumen of aorta with a plier curved end, and the denudation was verified by the absence of any degree of relaxation caused by carbachol (10^−4^ M), then, AcEO (10-4–10-1 mg/ml) was cumulatively added. In another set of experiments, endothelium-intact rings were pre-incubated with the muscarinic receptor antagonist atropine (1 μM; *n* = 6) and Ca^2+^-Calmodulin binding to NOS blocker calmidazolium chloride (10^−3^μM; *n* = 6) for 20 min prior the contraction with Phen (1 μM), then, the cumulative concentration–response curves of AcEO were constructed and compared with those obtained with untreated rings.

### Vasorelaxant effect of AcEO on aorta preincubated with L-NAME, Hydroxycobalamin, ODQ and 8-RP-Br-PET-cGMP

The endothelium-dependent vasorelaxant pathway was studied as described by Monteiro et al. [53]. Endothelium-intact rings were pre-incubated with the NO synthase inhibitor, L-NAME (10^−4^ M; *n* = 6), the NO scavenger, hydroxocobalamin (3.10^−5^ M; *n* = 6), the guanylyl cyclase inhibitor, ODQ (10^−5^M; *n* = 6), and the competitive cGMP-dependent protein kinase G (PKG) inhibitor, Rp-8-Br-PET-cGMP (3.10^−6^ M; *n* = 6) for 20 min prior the contraction with Phen (1 μM), then, the cumulative concentration–response curves of AcEO were constructed and compared with those obtained with untreated rings.

### Vasorelaxant effect of AcEO on aorta preincubated with potassium channels blockers, TEA, 4-AP, BaCl2 and Glybenclamide

The involvement of potassium channels in the vasorelaxant effect was assessed following the experimental procedure previously detailed, with some variations [54]. Endothelium-intact rings were incubated with the Ca^2+^-activated potassium channels, TEA (10^−2^M; *n* = 6), the selective voltage-activated potassium channel (K_*v*_) blocker, 4-AP (10^−4^M; *n* = 6), the selective inwardly-rectifying potassium channel blocker, BaCl_2_ (10^−4^M; *n* = 6), and the selective ATP-sensitive potassium channel blocker, glybenclamide (10^−5^M; *n* = 6) for 20 min prior to contraction with Phen (1 μM), then, the cumulative concentration–response curves of AcEO were constructed and compared with those obtained with untreated rings.

### Vasorelaxant effect of AcEO on aorta preincubated with Indomethacin, Thapsigargin and Verapamil

Calcium channels and prostanoid mediated vasodilation was studied following the protocol detailed by Li et al. [55]. Endothelium-intact rings were pre-incubated with the non-selective cyclooxygenase inhibitor, indomethacin (10^−5^M; *n* = 6), and to explore the role of calcium channels in the vasorelaxant effect, endothelium-intact rings were incubated with the Ca^2+^-channel type VOC, verapamil (10^−5^ M; *n* = 6) and the endoplasmic reticulum Ca^2+-^ATP_ase_ (SERCA) inhibitor, thapsigargin (10^−7^ M; *n* = 6) for 20 min prior to contraction with phenylephrine (10^−6^ M), then, the cumulative concentration–response curves of AcEO were constructed and compared with those obtained with untreated rings.

### Vasorelaxant effect of AcEO on aorta precontracted with Phen (1 μM) and K^+^ (80 mM) and comparative vasorelaxant effect of cumulative doses of ACEO and Verapamil on K^+^ (80 mM)

To confirm the involvement of voltage operated, and /or receptor operated calcium channels, and the enhancement of calcium release form internal stores in the vasodilator effect of AcEO, two sets of experiments were carried out as described by [56]. First, cumulative doses of the AcEO (0.1–100 μg/ml) were added to aorta precontracted with both K^+^ (80 mM) and Phen (1 μM). In another set of experiments, a comparative profile of the vasorelaxant effect of ACEO cumulative doses (10^−4^–10^−1^ mg/ml) and verapamil (10^−8^-10^−6^ M) was challenged on the aorta precontracted by K^+^ (80 mM).

### Statistical analysis

The data were expressed as the mean ± standard error of mean (SEM). The results were analyzed using one-way and two-way analysis of variance (ANOVA), followed by Bonferroni’s as a post-test. A value of *p* < 0.05 was considered significant. The linear and nonlinear regression tests have been used as well. The chemical structures have been drawn by using the freeware version of the software ACD/ChemSketch (Freeware) 14.01.

## Results

### Chemical analysis

**Fig. 1:**
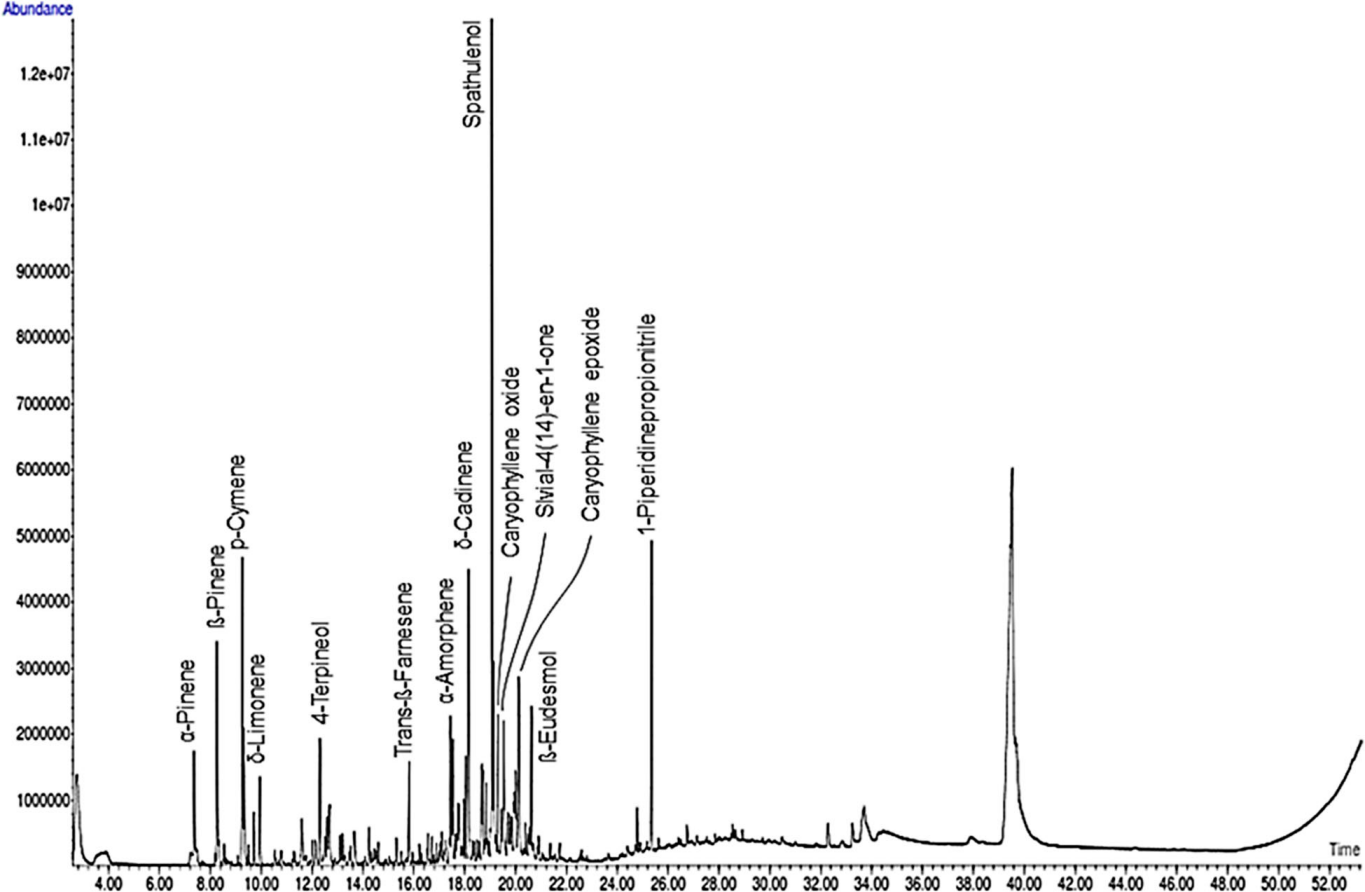
GC-MS chromtogram of *A. campestris* L. essential oil (AcEO)

**Fig. 2:**
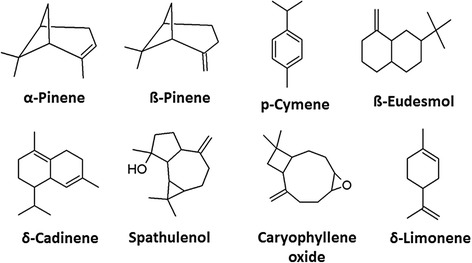
Chemical structures of volatiles compounds of *A. campestris* L. essential oil (AcEO)

Chemical analyses (GC and GC/MS) of AcEO allowed the identification of 42 compounds. The chromatographic profile of the essential oil is shown in (Fig. 1). The oil has the spathulenol (10.19%) as main component, followed by ß-eudesmol (4.05%), p-cymene (3.83%), δ-cadinene (3.67%), ß-pinene (2.82%), caryophyllene oxide (2.30%) and salvial-4(14)-en-1-one (2.51%). The compounds mentioned in Fig. 1 were systematically found in all the samples. The chemical structures of the major chemical components found in AcEO are presented in (Fig. 2).

### β-Carotene bleaching assay

**Fig. 3:**
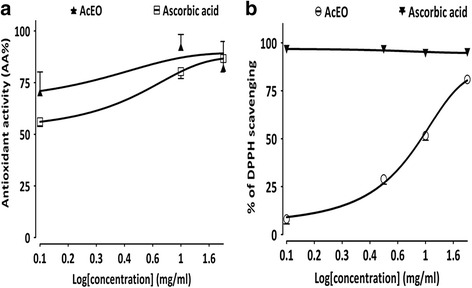
Antioxidant effect of *A.campestris* L. essential oil (AcEO) and ascorbic acid on (**a**) the scavenging of 2, 2(diphenyl-1-picryhydrazyl (DPPH) radical (**b**) the prevention of β-carotene bleaching and; Values are mean ± SEM, *n* = 3, and analyzed with linear regression test

The prevention of β-carotene bleaching with linoleic acid was similarly effective for AcEO (AA_max_% = 82.2% ± 12.7) and the standard ascorbic acid (AA _max_ % = 86.65% ± 6.45) and the antioxidant effect of both tested substrates was higher than 50% (Fig. 3a).

### DPPH radical scavenging assay

The radical scavenging activity of AcEO against DPPH radical increased significantly in dose-dependent manner (Fig. 3b); the EC_50_ values calculated from the graph was EC_50_ = 690 μg/ml. However, the radical scavenging activity attributed to ascorbic acid seems to be more important, characterized by a linear and stable shape of the graph with a maximum of 96.23% of the antioxidant effect (AA % higher than 50%).

### Acute toxicity

During the experiment, animals treated with AcEO at 2 g/kg, showed several signs of intoxication like: hyperactivity succeeded by asthenia, tremors, convulsion and irregular breathing, yet, all these signs disappeared after the first 24 h. The same dose provoked minimal lethality (one animal dead), marked by an intense ulceration of gastric mucosa. However, no significant changes have been observed in organs and body weights monitored during 2 weeks succeeding AcEO injection.

### Antiplatelet effect

**Fig. 4:**
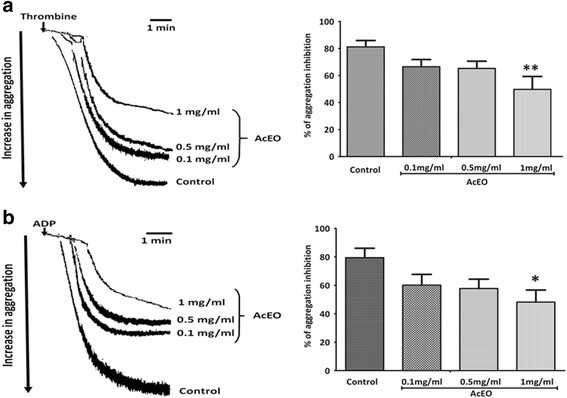
The originaltrcing and percentage of aggregation inhibition of 0.1, 0.5 and 1 mg/ml of *A. campestris* L. essential oil (AcEO) on thrombin (**a**) and ADP (**b**) induced platelet aggregation. Values are mean ± SEM, *n* = 6, analysed with one way ANOVA followed by Bonferroni’s post-test; p* < 0.005 vs control and ** < 0.01 vs control

AcEO (0.1, 0.5 and 1 mg/ml) added to washed platelets inhibited aggregation triggered by thrombin (0.1 U/ml) and ADP (1 μM). The dose 1 mg/ml showed a maximum inhibitory effect of, respectively, 49.73% ± 9.54 (*p* < 0.01) and 48.20% ± 8.49 (*p* < 0.05) on thrombin and ADP -induced platelet aggregation. However the doses 0.1 mg/ml and 0.5 mg/ml of AcEO have no significant effect on the antiplatelet effect (Fig. 4).

### Determination of the mechanism underlying the vasorelaxant activity of AcEO

#### Vasorelaxant effect of AcEO on denuded aorta, and on intact aorta preincubated with Atropine and Calmidazolium

**Fig. 5:**
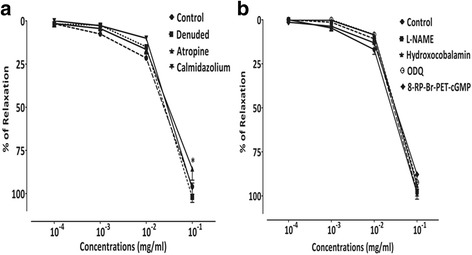
Concentration–response curves of the vasorelaxant effect of *A. campestris* L. (AcEO) on (**a**) isolated aorta pre-contracted with phenylephrine 10^−6^ M, on denuded aorta and on aorta pre-incubated with Atropine and Calmidazolium, (**b**) with L-NAME, Hydroxocobalamin, ODQ and 8-RP-Br-PET-cGMP. Values are mean ± SEM, *n* = 6, and analyzed with two way ANOVA followed by Bonferroni’s post-test; * *p* < 0.05 vs control

AcEO (0.1–100 μg/ml) abolished the contraction induced by phenylephrine on intact thoracic aorta, and induced 95.97% ± 2.03 of vasorelaxation. The vasorelaxant effect induced by AcEO on denuded aorta (102.07% ± 3.01; *p* > 0.05), also, the pretreatment with calmidazolium (97.07% ± 3.29; *p* > 0.05), blocker of calcium-calmodulin binding to NO synthase, did not affect the vasorelaxation induced by AcEO. However, the vasorelaxation provoked by AcEO on aorta precontracted with phenylephrine has been partially inhibited, when preincubated with the muscarinic receptor inhibitor, atropine (85.69% ± 6.56; *p* < 0.05) (Fig. 5a).

#### Vasorelaxant effect of AcEO on aorta preincubated with L-NAME, Hydroxycobalamin, ODQ and 8-RP-Br-PET-cGMP

No significant difference on the vasorelaxation effect of AcEO (0.1–100 μg/ml) on phenylephrine precontracted aorta have been observed in the presence of NO synthase inhibitor; L-NAME (97.27% ± 2.82; *p* > 0.05), in the presence of NO scavenger; hydroxocobalamin (93.99% ± 2.32; *p* > 0.05), and in the presence of guanylyl cyclase inhibitor; ODQ (92.26% ± 3.91; *p* > 0.05). However, relative inhibitory effect AcEO-induced vasorelaxation have been observed in the presence of the competitive cGMP-dependent protein kinase G (PKG) inhibitor; Rp-8-Br-PET-cGMP (87.86% ± 4.28; *p* < 0.05) (Fig. 5b).

#### Vasorelaxant effect of AcEO on aorta preincubated with potassium channels blockers, TEA, 4-AP, BaCl2 and Glybenclamide

**Fig. 6:**
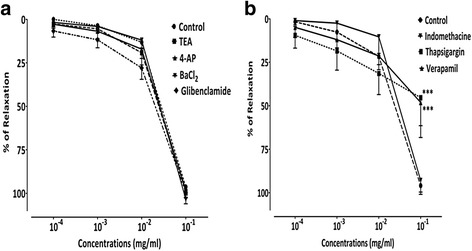
Concentration–response curves of the vasorelaxant effect of *A. campestris* L. (AcEO) on (**a**) isolated aorta pre-contracted with Phen 1 μM, and in the presence of TEA, 4-AP, BaCl_2_ and Glibenclamide, and in the presence of (**b**) Thapsigargin, Verapamil and Indomethacin. Values are mean ± SEM, *n* = 6, and analyzed with two way ANOVA followed by Bonferroni’s post-test; *** *p* < 0.001 vs control

Pretreatment of aorta with potassium channels blockers; TEA (98.91% ± 3, 73; *p* > 0.05), 4-AP (98.05% ± 2.96; *p* > 0.05), BaCl_2_ (103.52% ± 2.47; p > 0.05) and glybenclamide (99.55% ± 2.66; *p* > 0.05), showed no significant changes in AcEO (0.1–100 μg/ml) induced-vasorelaxation (Fig. 6a).

#### Vasorelaxant effect of AcEO on aorta preincubated with Indomethacin, Thapsigargin and Verapamil

**Fig. 7:**
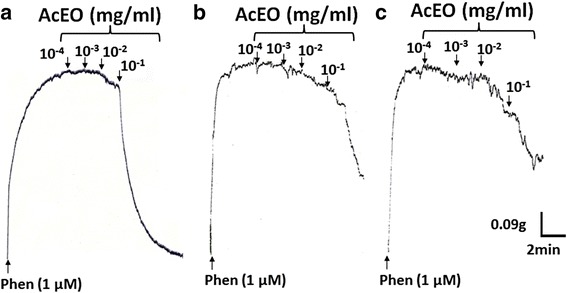
Original tracing showing the vasorelaxant effect of *A. campestris* L. essential oil (AcEO) on isolated aorta pre-contracted with Phen 1 μM (**a**) and in the presence of Verapamil (**b**) and Thapsigargin (**c**)

Pretreatment of aorta with cyclooxygenase inhibitor; indomethacin (92.95% ± 2.73; *p* > 0.05) did not produce any significant difference of vasorelaxation induced by AcEO precontracted with phenylephrine. Therefore, aorta preincubated with endoplasmic reticulum calcium-ATPase inhibitor; thapsigargin (45.34% ± 6.58; *p* < 0.001), and calcium channel type VOC blocker; verapamil (48.12% ± 8.21; *p* < 0.001) produced a sub-maximum inhibitory effect of vasorelaxation induced by AcEO (0.1–100 μg/ml) (Figs. 6b and 7).

#### Vasorelaxant effect of AcEO on aorta precontracted with Phen (1 μM) and K^+^ (80 mM) and comparative vasorelaxant effect of cumulative doses of ACEO and Verapamil on K^+^ (80 mM)

**Fig. 8:**
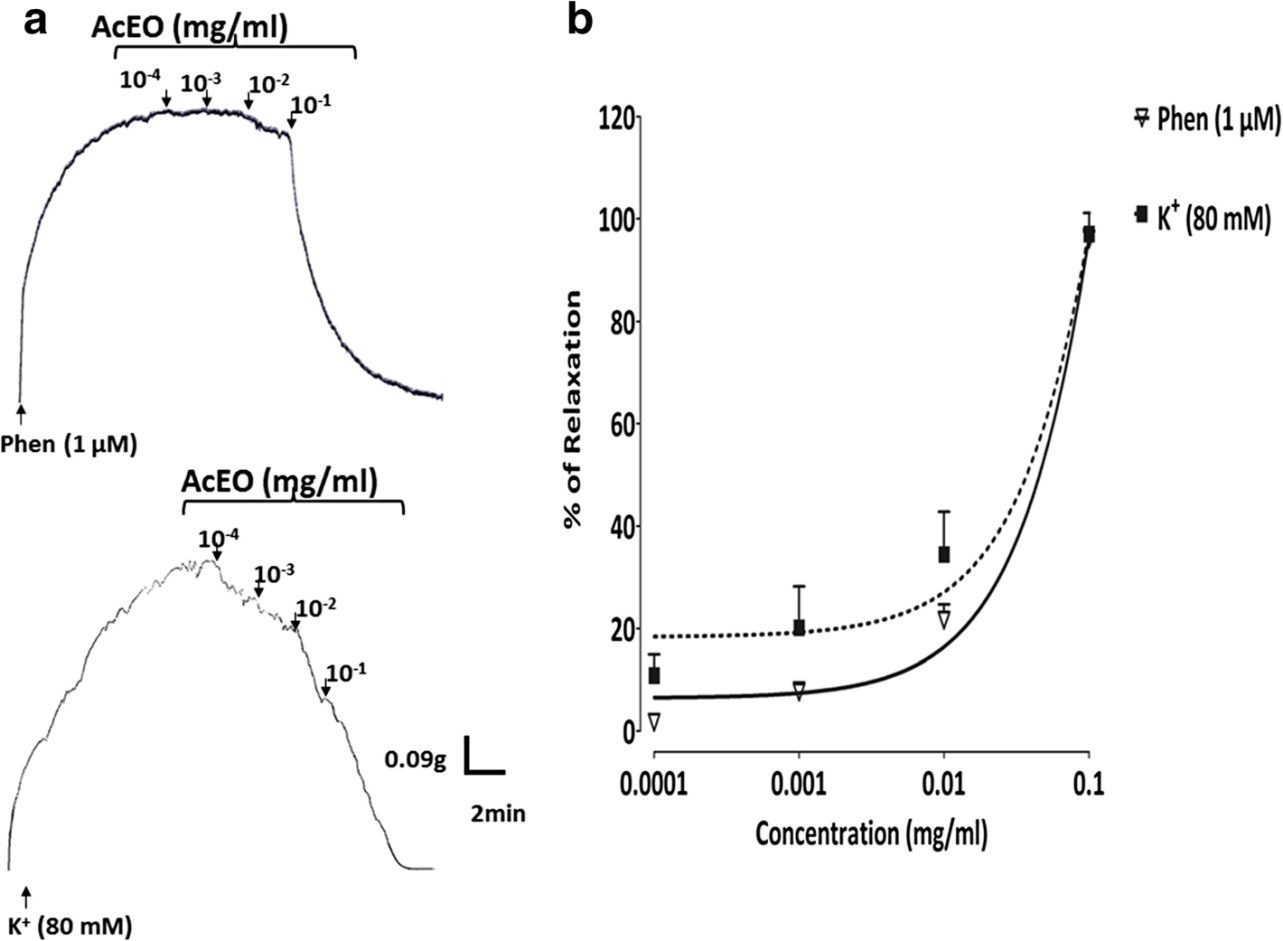
The originaltrcing (**a**) and the concentration–response curves (**b**) of the vasorelaxant effect of *A. campestris* L. essential oil (AcEO) on Phen (1 μM) and on K^+^ (80 mM)-induced contraction. Values are mean ± SEM, *n* = 6, and analyzed with non linear regression test

**Fig. 9:**
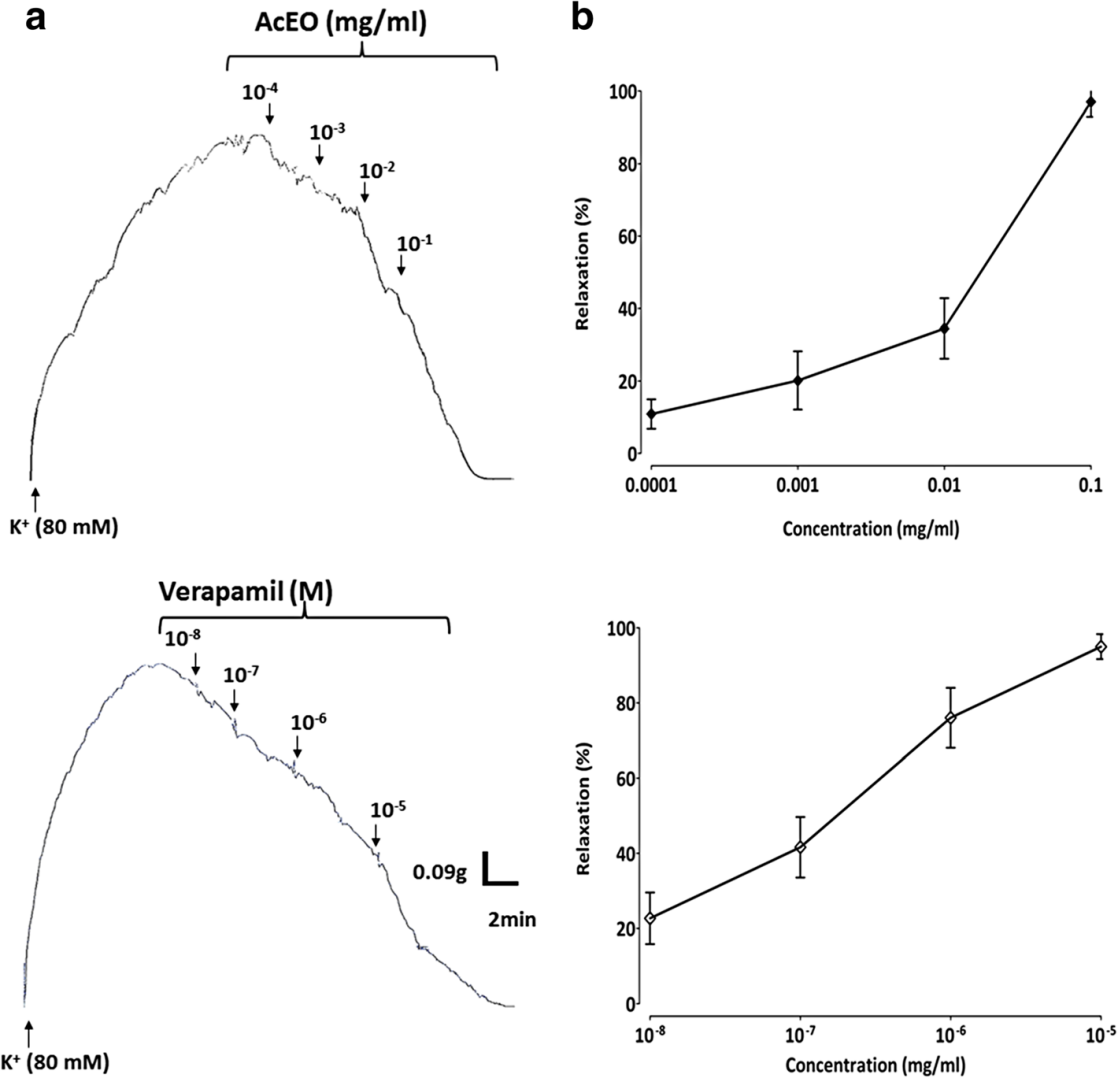
The original tracing (**a**) and the concentration–response curves (**b**) of the vasorelaxant effect of *A. campestris* L. essential oil (AcEO) and Verapamil on K^+^ (80 mM)-induced contraction. Values are mean ± SEM, *n* = 6, and analyzed with linear regression test

As shown in Fig. 8, AcEO triggered a concentration-dependent vasodilation against Phen (1 μM) and K^+^ (80 mM) induced vasocontractions, with respective EC_50_ value of 0.005 mg/ml (*n* = 6) and 0.019 mg/ml (*n* = 6). In parallel, AcEO and verapamil administered in increasing doses to the plateau of K^+^ (80 mM), showed a similar vasorelaxant profile with respective E_max_ values of (97,04% + 4,08; *n* = 6) and (94,97% + 3,35; *n* = 6) (Fig. 9).

## Discussion

Many studies have reported the chemical composition of essential oil of *A. campestris* L. growing in different countries. However, this study represents the first report about the essential oil of *A. campestris* L. (AcEO) existing in Morocco. As shown in Fig. 1, GC/MS analysis of essential oil of *A.campestris* L. resulted on the determination of spathulenol (10.19%) as the most prominent compound, followed by ß-eudesmol (4.05%), p-cymene (3.83%), δ-cadinene (3.67%), and ß-pinene (2.82%). These findings are consistent with our new published paper, which confirmed the similar chemical profile of *A.campestris* L. essential oil collected in the flowering stage of the year 2014 [57]. By comparison to the literature data, our findings are partially in accordance with a study conducted in Iran by Kazemi et al. [58], in which the predominated components of essential oil obtained from the flowers, leaves and stems of *A.campestris* L. were *α*-pinene (23–29.2%) and spathulenol (15.8–29.2%). Another work revealed the existence of the major volatiles spathulenol and *β*-pinene in essential oil of *A. campestris* L. from Serbia [2]. Previous studies reported the existence of two chemotypes of *A. campestris* L. essential oil occurring in different localities; the most relevant chemotype consists mainly in *β*-pinene alone or together with *α*-pinene occurring mainly in Tunisia [14, 59–62], Algeria [10, 63–65], and Southern Ural [66], while the other chemotype was characterized by the volatiles: tremetone and capillen, detected in essential oil of *A. campestris* L. growing in Turkey [24]. Moreover, it is known that the species *A.campestris* L. presents great variability in its essential oil composition due to the existence of different subspecies from different localities. In France, *A.campestris* subsp. glutinosa has the main terpenes: γ-terpinene and capillene [27, 67], while, the same subspecies from Italy presented the major compounds: *β-*pinene, germacrene D and bicyclogermacrene [68]. Other studies reported that *A.campestris* subsp. campestris essential oil from Lithuania and Poland possesses the main components: caryophyllene oxide, Z-falcarinol, germacrene D, β-pinene, γ-curcumene, γ-humulene [69–72]. The oils of other subspecies, maritima from Portugal and borealis from Italy were rich in *β-*pinene, cadin-4-en-7-ol, caryophyllene oxide and *α*-pinene [73–75]. Taking into account all these data, the species *A. campestris* L. can be segregated into many chemotypes based on the variation of the essential oil composition, which may lead to consider the species growing in the arid region of Eastern Morocco as a new chemotype characterized by the spathulenol as the major compound.

Antioxidant effect of AcEO evaluated on the scavenging of DPPH radical (EC_50_ = 690 μg/ml) seems to be considerable, though, the absence of a dose-response effect with both AcEO and ascorbic acid seems to be confusing. Indeed, the DPPH test reproducibility is controversial, since it is limited by its lack of specificity; the DPPH assay is not a competitive reaction, because the purple color of the DPPH radical can be easily lost via either hydrogen atom transfer (HAT) or reduction through single electron transfer (SET). Otherwise, the steric accessibility of DPPH radical represent the major limitation of this test that confers a more accessibility of the small molecules to the radical site and which may consequently have a higher antioxidant effect. On the other hand, a large brand of antioxidant compounds with peroxyl radicals may react slowly or may even be inert in reaction to DPPH which is a nitrogen radical in the first place [76, 77]. All these artefacts may interfere with the actual antioxidant effect of the AcEO and the ascorbic acid used as reference, which may blind their effective antioxidant effect and omit the dose-response efficiency of both substrates. On the other hand, the DPPH radical scavenging obtained with AcEO seems to be very important when compared to that observed with the essential oil obtained from the aerial part (IC_50_ = 94500 μg/ml) [14] and leaves (IC_50_ = 1874 μg/ml) of Tunisian *A.campestris* L. [59]. However, the essential oil extracted from leaves of *A.campestris* L. occurring in Algeria appears to have more efficient antioxidative potential on DPPH radical (IC_50_ = 39 μg/ml) [17]. On the other hand, AcEO prevented 82.2% of β-carotene bleaching with lineolate substrate, which is in agreement with study reported by Akrout et al. [14].

The oral acute toxicity of AcEO resulted on an LD_50_ value greater than 2 g/kg, body weight. The essential oil caused a minimal lethality witnessed by one dead animal in the group fed by 2 g/kg, body weight, and which was marked with an intense perforations and ulcerations of gastric mucosa that probably causes the death. Otherwise, there were any adverse effects on the body weight or the organs weights monitored during the 15 days of the study. On the basis of the present data AcEO is considered as toxic at 2 g/kg due to gastric lesions induced. Additional studies about the toxicity of *A. campestris* L. are available, showing that the intraperitoneal administration of aqueous extract to mice showed an earlier toxicity with an LD_50_ = 2.5 g/kg of body weight [39]. Furthermore, an acute toxicity test of the essential oil of *A.campestris* L. on brine shrimp larvae (*Artemia* sp.) gave the median lethality dose ranging from 15 to 20 μg/ml [69]. Concerning the platelet aggregation induced by both agonists thrombin (0.1 U/ml) and ADP (1 μM), AcEO was able to reduce it about 50%; so it can be postulated that this oil may interact with the site of action of ADP and thrombin and hence interrupting their cellular signaling and antagonizing the aggregation process. This results seems to be very considerable, by reference to a previous study conducted by our team, where the pharmaceutical anti-aggregant reference acetylsalicylic acid (1 mg/ml) induced a total inhibition of the aggregability caused by thrombin but at a quite high dose (1 mg/ml), if compared with our study when thrombin is tested at the dose 0.1 mg/ml [78]. It has been reported that platelet activation and aggregation are participating in the emergence of hypertension in different ways [79]. Platelets activation in hypertension can be explained by several mechanisms, among which the auto-degranulation that leads to activation of platelets exposed to increased shear force as result of high blood pressure [80]. Activated platelets released endogenous mediators like ADP which is known as a platelet aggregating agent that interact with two platelets receptors: Gq-coupled P2Y_1_ that induces a transient rise in free cytoplasmic calcium and Gi-coupled P2Y_12_ that provoke inhibition of adenylyl cyclase. Both pathways are necessary to elicit platelet aggregation [81]. Thrombin is a another platelet agonist that enhance aggregation; in fact, thrombin induced platelets activation via protease-activated receptors (PARs) that has a protein Gq-action, enhancing activation of phospholipase C (PLCβ), which hydrolyzes phosphatidylinositol 4,5 bisphosphate (PIP_2_), that promote the production of second messenger IP_3_, which contributes to the increase in intracellular Ca^2+^ through mobilization from internal stores and influx from the extracellular department. The increase in intracellular Ca^2+^ regulates many events leading to platelet aggregation [81].

The vasorelaxant effect of AcEO is obvious, since it succeeded to abolish the contraction triggered by phenylephrine, and produced a complete relaxation of aorta. Indeed, it is well evidenced that phenylephrine stimulates vascular contractions by acting through stimulation of α_1_ adrenergic receptors, which will provoke the conversion of phosphatidylinositol to inositol 1, 4, 5-triphosphate, leading to the release of Ca^2+^ from the intracellular stores [82]. In light of these finding, we aimed to highlight the mechanism of action of this vasorelaxant effect, by exploring many cellular signaling mechanisms, including endothelium-dependent and independent pathways.

The endothelium is a highly specialized layer of luminal blood vessel, playing a key role in the vasorelaxation, mediated mainly by the release of endothelium-derived vasodilators, like nitric oxide (NO) and prostacyclin [83–86]. By reference to our data, AcEO vasorelaxant effect appears to be endothelium-independent, since the vascular response persisted after removal of the endothelium. In the endothelial cell, the signalling mechanism responsible for muscarinic receptor-dependent NO production involves Ca^2+^ and calmodulin-dependent activation of eNOS [87]. Once produced, calcium/calmodulin complex (Ca^2+^/CaM) enhance the dissociation of eNOS from caveolae, which become catalytically active and induces NO production [88]. The NO, as released by endothelial cells, increased cGMP levels in the smooth muscle, activated PKG, and phosphorylated the same vascular smooth muscle proteins, which induces a decrease in intracellular calcium concentration and a subsequent vasorelaxation [89]. To check up the involvement of this pathway in the vasorelaxant action of AcEO, aorta was submitted to specific inhibitors and blockers of endothelium mediators that triggered the vasorelaxant action like atropine (non-selective antagonist muscarinic receptors), calmidazolium (Ca^2+^-calmodulin binding to NOS blocker), L-NAME (NOS inhibitor), hydroxocobalamin (NO scavenger), ODQ (inhibitor of soluble guanylyl cyclase) and protein kinase G (PKG) inhibitor (Rp-8-Br-PET-cGMPs). Even though, the vasorelaxation was not affected in the presence of these drugs, which confirm that endothelium and specifically NO-GC-PKG pathway was not involved in this effect.

Another pathway of vasorelaxation endothelium-dependent has been studied; it’s about the COX product: PGI_2_ which is recognized for its potential ability to relax vascular smooth muscle [90] via activation of second messenger cAMP [91]. Hence, the possible role of PGI_2_ seems to be ruled out, because the pretreatment of aorta with, indomethacin, the COX inhibitor, did not change the vasorelaxant effect induced by AcEO.

In vascular smooth muscle cell membrane, the opening of potassium channels (K^+^) enhance an increase in K^+^ efflux, provoking membrane potential (*Em*) hyperpolarization and subsequent closure of voltage-activated calcium (Ca^2+^) channels, causing a decrease of intracellular Ca^2+^ mobilization followed by a vasodilation [92]. In our experiments, the treatment of aorta with potassium channels blockers did not change the vasodilator action induced by AcEO, which suggests that the observed effect is potassium channel-independent. Besides K^+^ channels, Ca^2+^ channels contribute to the relaxant effect of resistant vessels. The Ca^2+^ influx into vascular smooth muscle have two preponderant pathways: one is an L-type Ca^2+^ channel and the other is a store-operated Ca^2+^ channel (STOC). L-type calcium channels are the main gate of Ca^2+^ mobilization from extracellular space during cell excitation. The Ca^2+^ influx through L-type Ca^2+^ channels is the determinant of intracellular calcium level in the vascular smooth muscle and hence the key parameter of contraction [93]. Hence, the calcium antagonism via L-type calcium channel is a recognized as a mechanism of vasorelaxation [94]. A decrease in Ca^2+^ levels into sarcoplasmic reticulum (SR) triggers refilling of cytoplasmic Ca^2+^ in the SR Ca^2+^ store through sarco/endoplasmic reticulum Ca^2+^ ATPase (SERCA) pump and decreasing Ca^2+^ influx from STOC, which consequently decreases intracellular Ca^2+^ and enhance the vasorelaxation [95]. We performed separate experiments with the blockers of SERCA pump (thapsigargin) and L-type calcium channels (verapamil). As result, we found that AcEO-vasorelaxation induced was decreased by 50% with both drugs. In addition, AcEO inhibited KCL-induced contraction, and subsequently reduced the Ca^2+^-induced contraction in aortic rings exposed to KCl, and this effect was similar to that observed with increasing doses of verapamil, a known calcium channel blocker and a vasorelaxant drug, which confirms that AcEO acts by blocking the L-type calcium channels. However, *A.campestris* L. oil also inhibited Phen-induced contraction, suggesting that it attenuated Ca^2+^ influx through receptor-operated Ca^2+^ channels as well.

In the matter of fact, the antagonizing effect of verapamil and thapsigargin remains debatable, since a persistent vasorelaxation of AcEO was maintained after the pretreatment with both drugs. These results suggest that the vasodilator effect of AcEO may involve the synergetic contribution of both calcium channels; otherwise, AcEO may act concomitantly, on both channels, by blocking VOCC channels, and by activating the SERCA pump, both mechanisms will together participate in an additive and/or synergetic manner to decrease the intracellular levels of calcium leading to a subsequent vasorelaxation.

## Conclusion

Herein, we have shown that the chemical profile drawn for essential oil of *A. campestris* L. growing in Eastern Morocco reveal a new chemotype related to this region and spathulenol was identified as the predominant component of this oil. The pharmacological properties attributed to AcEO, like antioxidant, antiplatelet and vasorelaxant effects seem to be very interesting. The present work provide, also, evidence about the signaling mechanism of vasorelaxation induced by AcEO, showing that essential oil acts via L-type calcium channels and SERCA pumps to reduce intracellular calcium, and consequently triggering a sustained vasodilation.

## Abbreviations

4-AP: 4-Aminopyridine
AA: Antioxidative activity
Ac_0_: Control absorbance before incubation
Ac_120_: Control absorbance after 2 hours incubation
AcEO: *Artemisia campestris* L. essential oil
ADP: Adenosine diphosphate
As_120_: Sample absorbance after 2 hours incubation
ATP: Adenosine triphosphate
BaCl_2_: Barium chloride
Ca^2+^-CaM: Calcium-calmodulin complex
cAMP: Cyclic adenosine monophosphate
cGMP: Cyclic guanosine monophosphate
COX: Cyclooxygenase
DMSO: Diméthylsulfoxyde
DPPH: 2, 2-Diphenyl-1-picrylhydrazyl
EC_50_: Half maximal effective concentration
EC_max_: Effective concentration that gives the maximal effect
Em: Membrane potentiel
eNOS: Endothelial nitrous oxide synthase
GC: Guanylyl cyclase
GC-MS: Gas chromatography–mass spectrometry
Gq-coupled P2Y_1_: Purinergic receptor P2Y, G-protein coupled, 1
Gq-coupled P2Y_12_: purinergic receptor P2Y, G-protein coupled, 12
IP_3_: Inositol trisphosphate
Kv: Voltage activated potassium channel
L-NAME: Nω-Nitro-L-arginine methyl ester hydrochloride
MS: Mass spectrometry
NO: Nitrous oxide
NOS: Nitrous oxide synthase
ODQ: 1H-[1,2,4]Oxadiazolo[4,3-a]quinoxalin-1-one
PARs: Protease activated receptors
PGI_2_: Prostacyclin
Phe: Phenylephrine
PIP_2_: Phosphatidyl inositol diphosphate
PKG: Protein kinase G
PLCβ: Phospholipase Cβ
PRP: Platelet rich plasma
Rp-8-Br-PET-cGMPS: Rp-8-Bromo-β-phenyl-1,N2-ethenoguanosine 3′,5′-cyclic monophosphorothioate sodium salt
SERCA: Sarco/endoplasmic reticulum Ca^2+^-ATPase
SR: Sarcoplasmic reticulum
STOC: Store operated calcium channel
TEA: Tetraethylammonium
VOC channels: Voltage-operated calcium channels.
